# Association between gestational trophoblastic disease (GTD) history and clinical outcomes in in vitro fertilization/intracytoplasmic sperm injection (IVF/ICSI) cycles

**DOI:** 10.1186/s12958-022-00898-2

**Published:** 2022-02-04

**Authors:** Xinyu Cai, Mei Zhang, Chenyang Huang, Yue Jiang, Jidong Zhou, Manlin Xu, Guijun Yan, Haixiang Sun, Na Kong

**Affiliations:** 1 Reproductive Medicine Center, Nanjing Drum Tower Hospital, The Affiliated Hospital of Nanjing University Medical School, Nanjing, 210008 Jiangsu China; 2 Reproductive Medicine Center, Nanjing Drum Tower Clinic Medical College of Nanjing Medical University, Nanjing, 210008 Jiangsu China

**Keywords:** IVF/ICSI, GTD history, live-birth rate, gestational age at delivery, endometrial receptivity

## Abstract

**Background:**

Gestational trophoblastic disease (GTD) usually affects young women of childbearing age. After treatment for GTD, 86% of women wish to achieve pregnancy. On account of the impacts of GTD and treatments as well as patient anxiety, large numbers of couples turn to assisted reproductive technology (ART), especially in vitro fertilization/intracytoplasmic sperm injection (IVF/ICSI). But few studies have investigated whether a history of GTD affects the outcomes of IVF/ICSI in secondary infertile patients and how it occurs. We investigate whether a history of GTD affects the IVF/ICSI outcomes and the live birth rates in women with secondary infertility.

**Methods:**

This retrospective cohort study enrolled 176 women with secondary infertility who underwent IVF/ICSI treatment at the reproductive medical center of Nanjing Drum Tower Hospital from January 1, 2016, to December 31, 2020. Participants were divided into the GTD group (44 women with GTD history) and control group (132 women without GTD history matched from 8318 secondary infertile women). The control group and the study group were matched at a ratio of 3:1 according to patient age, infertility duration, number of cycles and body mass index (BMI). We assessed retrieved oocytes and high-grade embryos, biochemical pregnancy, miscarriage, ectopic pregnancy, gestational age at delivery, delivery mode and live birth rates.

**Result(s):**

We found a significantly reduced live-birth rate (34.1% vs 66.7%) associated with IVF/ICSI cycles in patients with a GTD history compared to those without a GTD history. The biochemical pregnancy and miscarriage rates of the GTD group were slightly higher than those of the control group. In addition, there was a difference in gestational age at delivery between the GTD and control groups (*p* < 0.001) but no differences in the mode of delivery (*p* = 0.267). Furthermore, the number of abandoned embryos in the GTD group was greater than that in the control group (*p* = 0.018), and the number of good-quality embryos was less than that in the control group (*p* = 0.019). The endometrial thickness was thinner (*p* < 0.001) in the GTD group. Immunohistochemistry (IHC) showed abnormal endometrial receptivity in the GTD group.

**Conclusion(s):**

The GTD history of patients undergoing IVF/ICSI cycles had an impact on the live-birth rate and gestational age at delivery, which might result from the thinner endometrium and abnormal endometrial receptivity before embryo transfer.

**Supplementary Information:**

The online version contains supplementary material available at 10.1186/s12958-022-00898-2.

## Introduction

Gestational trophoblastic disease (GTD) covers a range of premalignant and malignant pregnancy-related disorders associated with highly abnormal placental trophoblastic tissue [1]. GTD includes 5 histological types: hydatidiform mole, erosive hydatidiform mole, choriocarcinoma, placental site trophoblastic tumor (PSTT) and epithelioid trophoblastic cell tumors [2].

Although GTD was previously considered a lethal disease, progressive improvements in the diagnostic process, clinical management and follow-up protocols as well as the development of effective treatments and centralization of care have resulted in excellent cure rates (exceeding 98%) [3]. Future reproductive outcomes are important because trophoblastic diseases of pregnancy usually affect young women of childbearing age. Most GTD patients can subsequently achieve natural pregnancies, and large-sample retrospective studies have found that the natural pregnancy outcome is similar to that of the general population [4]. However, one study has revealed that the proportion of secondary infertility in GTD patients is approximately 4.4% [5].

Treatments for GTD usually include uterine curettage and chemotherapy, both of which have a negative influence on women’s fertility and psychological states. Even though GTD evolves from a previous pregnancy, not all women are interested in a new pregnancy, and some may avoid this due to treatment-related anxiety. After treatment for GTD, 86% of women wish to achieve pregnancy [6]. Because of the impacts of GTD and treatments as well as patient anxiety, large numbers of couples turn to assisted reproductive technology (ART), especially in vitro fertilization/intracytoplasmic sperm injection (IVF/ICSI).

The outcomes of IVF/ICSI in secondary infertile patients with a history of GTD remain unknown. There are few reports of IVF/ICSI in infertile patients with a history of GTD, and only individual cases have been reported. Pal et al. [7] reported that one patient with a history of hydatidiform mole received IVF/ICSI treatment because of fallopian tube blockage. They found that the abnormal fertilization rate and triploid embryo rate were significantly higher and that the patient could not achieve a successful pregnancy after a total of 9 embryos were transferred. Few studies have investigated whether a history of GTD affects the outcomes of IVF/ICSI in secondary infertile patients. Therefore, we investigated patients with a GTD history who received IVF/ICSI treatments at our reproductive medical center from January 2016 to December 2020 to analyze the live-birth rates and other clinical outcomes associated with IVF/ICSI cycles; we compared these patients with secondary infertile patients without a previous GTD history.

## Materials and methods

### Study population and design

**Fig. 1:**
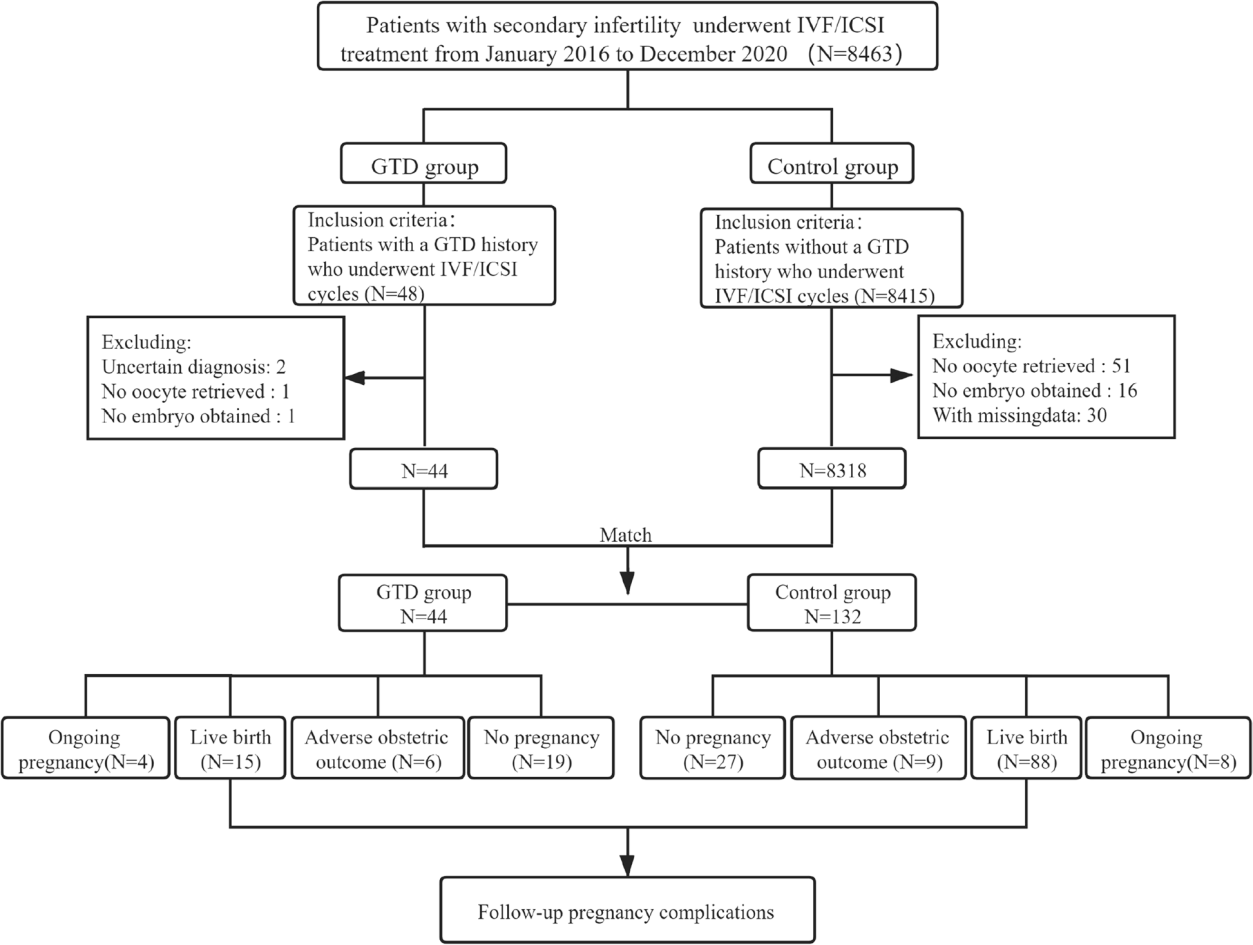
Flowchart of the participant selection and follow-up procedure throughout the study. Abbreviation: IVF/ICSI, in vitro fertilization/intracytoplasmic sperm injection; GTD, gestational trophoblastic disease

All secondary infertile patients undergoing IVF/ICSI treatments from January 1, 2016, to December 31, 2020, at the reproductive medical center of Nanjing Drum Tower Hospital were reviewed (Fig. 1). Patients with a GTD history were included in the study group, and secondary infertile patients without a GTD history were included in the control group. We excluded cases of hydatidiform mole with uncertain diagnosis and tubal hydatidiform mole pregnancy. A total of 44 patients were involved in the study group. The control group and the study group were matched at a ratio of 3:1 according to patient age (±1 year), infertility duration (±1 year), number of cycles (±1) and body mass index (BMI) (±0.5 kg/m^2^) with Social Sciences Statistical Package (SPSS). This study was approved by the Ethics Committee of Drum Tower Hospital, which is affiliated with the Nanjing University Medical School (no. 2021–104-01).

### Clinical protocols

#### Controlled ovarian hyperstimulation (COH)

According to age, weight, ovarian reserve, and prior response to stimulation, all the patients underwent controlled ovarian hyperstimulation (COH) with a flexible protocol for the medication dose selected. When the diameters of at least two follicles reached 16–18 mm, ovulation was triggered. Subcutaneous injection of 0.2 mg gonadotropin-releasing hormone agonist (GnRHa, Decapeptyl) and/or intramuscular injection of 5000–10,000 IU human chorionic gonadotropin (hCG) was given [8]. After 36 to 38 h, oocytes were retrieved using transvaginal puncture. On the day of retrieval, oocytes were mechanically denuded to assess nuclear maturity and underwent standard insemination or intracytoplasmic sperm injection (ICSI) according to male sperm parameters [9–11].

#### Embryo culture and transplantation

Four to six hours after oocyte retrieval, treated sperm were added for IVF or ICSI. Fertilization was defined as the presence of two pronuclei the morning after oocyte retrieval. After 2–3 days of in vitro culture, the quality of the embryos was evaluated, and high-quality embryos were selected for transfer or freezing [12, 13], The criteria for good-quality embryos were normal development rate, such as 4 cells on day 2 and 8–10 cells on day 3, and an embryonic morphology score of 3–4 [14, 15]. Some embryos were cultured to the blastocyst stage. Blastocyst formation was determined by progression to the blastocyst stage on days 5, 6 or 7 [16]. For patients who opted to use preimplantation genetic testing for aneuploidy (PGT-A), the embryos were biopsied at the blastocyst stage. The fertilization rate is the ratio of the number of fertilized embryos to the number of mature oocytes retrieved, while the normal fertilization rate is the ratio of the number of normally fertilized embryos to the number of mature oocytes retrieved.

Frozen embryo transfer (FET) cycle: The hormone replacement cycle started on the second day of menstruation by 14–20 days of estradiol pills. When the endometrial thickness reached at least 8 mm, progesterone was injected for 5–6 days, and then the embryos were transferred. Follicular development and LH values were monitored during the natural cycle, and embryo transfer was performed on the 4th/5th day after the LH peak or the 3rd/4th day after ovulation.

One or two good-quality embryos were selected and transferred to the uterus through abdominal ultrasound guidance. After embryo transfer, oral dydrogesterone or estradiol combined with dydrogesterone was employed with vaginal progesterone for luteal support. Serum β-hCG was detected 14 days after embryo transfer. If serum β-hCG was positive (> 200 mIU/mL), biochemical pregnancy was confirmed. Transvaginal ultrasound examination was performed 28–30 days after embryo transfer, and clinical pregnancy was determined by the presence of the gestational sac. Outcome data were collected until the live birth.

### Observational target and outcomes

The general fertility of each patient was determined based on age, infertility duration, BMI, antral follicle count (AFC), and levels of follicular stimulating hormone (FSH), luteinizing hormone (LH), and estrogen (E_2_). The process of ovulation induction was mainly reflected as the days of ovulation hyperstimulation, the amount of Gn, the level of E_2_ on the trigger day and the number of oocytes retrieved. The main results of ovulation induction were the number of oocytes retrieved, fertilization rate, normal fertilization rate, number of good-quality embryos, and abandoned embryos. Clinical outcomes were the implantation rate (the ratio of total implantation embryo number to total number of transferred embryos), miscarry rate (the ratio of miscarry number to the number of clinical pregnancies), live-birth rate, gestational age at delivery and cesarean delivery rate.

### Statistical analysis

We checked differences between groups using parametric and nonparametric descriptive statistics. Multivariate logistic regression was conducted to identify variables independently associated with the live-birth outcome. The primary exposure factor was defined as a history of GTD. The covariates were as follows: age (< 35 vs ≥35 years old), infertility duration (< 3 vs ≥3 years), gravidity (< 3 vs ≥3), number of uterine curettages (< 1 vs ≥1), type of ART (IVF vs ICSI vs PGT), endometrial thickness on trigger day (≤9 mm vs 9–12 mm vs ≥12 mm), number of good-quality embryos (< 3 vs ≥3), and number of abandoned embryos (< 6 vs ≥6). Data analysis was performed using EmpowerStats 2.0 and R statistical software.

### Samples and immunohistochemical (IHC)

After ethical approval and written informed consent, we used residual endometrial biopsy samples collected during the hysteroscopic surgery of patients with suspected endometrial polyp、adhesion or endometritis but proved without later in both Control and GTD group. The dewaxed hydrated paraffin-embedded tissue sections were immersed in 3% H_2_O_2_ and 100% methanol for 30 min at room temperature to quench endogenous peroxidase, embedded in partial segments with 10% normal goat serum, and incubated with anti-FHL1 (1:500 dilution, MAB5938, SR&D Systems), anti-FOXO1 (1:500 dilution, 2880S, Cell Signaling Technology), anti-HOXA10 (1:500, sc-271,428, Santa Cruz Biotechnology), or anti-Ki67 (1:1000 dilution, ab15580, Abcam) overnight at 4 °C. On the following day, the sections were incubated with secondary antibody conjugated to horseradish peroxidase (Vector Laboratories) for 1 h at room temperature. Immunoreactivity was measured using diaminobenzidine (DAB; Vector Laboratories), photographed using microscopy software, and then analyzed using Image-Pro.

## Results

In total, 176 IVF/ICSI cycles from January 1, 2016, to December 31, 2020 met the inclusion criteria, 44 patients had a history of GTD, and 132 patients did not have a GTD history. The patients in our cohort study were 31.6 ± 3.9 years old. The infertility duration was 3.1 ± 2.5 years, and tubal factor (63.6%) was the most common infertility diagnosis.

### Baseline clinical characteristics

In all, 8318 cycles with secondary infertility were included in the statistics before matching, and the data regarding the major clinical characteristics are presented in Table 1. According to patient age (±1 year), infertility duration (±1 year), number of IVF/ICSI cycles (±1) and BMI (±0.5 kg/m^2^), we matched 132 cycles associated with patients with no history of GTD; these patients served as the control group. On account of the matching, there were no differences in patient age (31.9 ± 4.1 vs 31.2 ± 4.4, *p* = 0.297), infertility duration (3.3 ± 2.5 vs 2.5 ± 2.2, *p* = 0.056), number of IVF/ICSI cycles (1.0 (0.0–7.0) vs 1.0 (1.0–5.0), *p* = 0.106) or BMI (22.8 ± 3.3 vs 23.4 ± 3.2, *p* = 0.259) (Table 2). Idiopathic and genetic factors and unknown causes accounted for more infertility diagnoses in the GTD group than in the control group, but male factors, tubal factors and ovulatory dysfunction accounted for fewer infertility diagnoses. There were statistically significant differences in gravidity (1.0 (0.0–5.0) vs 2.0 (1.0–7.0), *p* < 0.001) and curettage times (0.0 (0.0–3.0) vs 2.0 (0.0–6.0), *p* < 0.001). In addition, we found no statistically significant differences in ovarian reserve reflected by AFC (15.7 ± 6.2 vs 16.0 ± 7.0, *p* = 0.770) and FSH (7.6 ± 3.0 vs 8.2 ± 5.2 mIU/mL, *p* = 0.257) (Table 2).

**Table 1:** Demographics of patients with gestational trophoblastic disease history (GTD group) or not (control group) before and after matching

Characteristic	Before Matching			After Matching		
	Control group (*n* = 8318)	GTD group (*n* = 44)	*P* value	Control group (*n* = 132)	GTD group (*n* = 44)	*P* value
Age (year) ^a^	31.9 ± 4.1	31.2 ± 4.4	0.297	31.8 ± 3.7	31.2 ± 4.4	0.432
Infertility duration (year) ^a^	3.2 ± 2.6	2.5 ± 2.2	0.047	3.3 ± 2.5	2.5 ± 2.2	0.056
Number of IVF/ICSI cycles ^b^	1.0 (0.0–17.0)	1.0 (1.0–3.0)	0.166	1.0 (0.0–7.0)	1.0 (1.0–5.0)	0.106
BMI (kg/m^2^) ^a^	23.1 ± 3.2	23.4 ± 3.2	0.520	22.8 ± 3.3	23.4 ± 3.2	0.259

^a^Values are mean ± standard deviation.

^b^Values are median and Min-Max.

**Table 2:** Clinical characteristics of GTD group and control group

Characteristic	Control group (*n* = 132)	GTD group (*n* = 44)	*P* value
Infertility diagnosis n (%)			< 0.001
Idiopathic	0 (0.0%)	2 (4.5%)	
Male factor	18 (13.6%)	3 (6.8%)	
Tubal factor	88 (66.7%)	24 (54.5%)	
Anovulatory	17 (12.9%)	4 (9.1%)	
Endometriosis	7 (5.3%)	2 (4.5%)	
PGT	0 (0.0%)	3 (6.8%)	
Unknown cause	2 (1.5%)	6 (13.6%)	
AFC ^a^	15.5 ± 6.7	16.0 ± 7.0	0.681
FSH (mIU/mL) ^a^	7.6 ± 3.0	8.2 ± 5.2	0.383
LH (mIU/mL) ^a^	5.1 ± 3.2	5.1 ± 2.3	0.984
E_2_ (pg/mL) ^a^	45.5 ± 45.8	35.2 ± 20.8	0.156
Gravidity ^b^	1.0 (0.0–5.0)	2.0 (1.0–7.0)	< 0.001
Parity ^b^	0.0 (0.0–1.0)	0.0 (0.0–1.0)	0.273
No. of curettage ^b^	0.0 (0.0–3.0)	2.0 (0.0–6.0)	< 0.001

^a^Values are mean ± standard deviation.

^b^Values are median and Min-Max.

*P* value: For continuous variables, it can be obtained by Kruskal Wallis rank sum test. If the count variable has a theoretical number < 10, it can be obtained by Fisher’s exact probability test.

### Ovulation induction and embryology laboratory outcomes

The mean days of ovarian stimulation of the GTD group were 10.0 (5.0–18.0) with a total gonadotropin dose of 1977.7 ± 897.5 IU and a total GnRHa dose of 1.2 mg (0.0–3.8), which were similar to those of the control group. In addition, E_2_ levels on the trigger day, number of mature oocytes retrieved, fertilization rate and normal fertilization rate were similar in the two groups (*p* > 0.05). There were 23 (52.3%) patients who underwent ICSI cycles and 6 (13.6%) patients who underwent PGT cycles in the GTD group, which were significantly more than those in the control group. Furthermore, we found that the number of good-quality embryos in the GTD group was less than that in the control group (3.5 ± 3.2 vs 4.6 ± 2.6, *p* = 0.019), but the number of abandoned embryos was much greater than that in the control group (4.0 ± 4.2 vs 2.8 ± 2.2, *p* = 0.018). The endometrial thickness on the trigger day was significantly thinner in the GTD group (control group: 11.5 ± 2.4 mm, GTD group: 9.3 ± 2.5, *p* < 0.001). All these results were shown in Table 3.

**Table 3:** Ovulation induction and embryology laboratory outcomes

Characteristics	Control group (*n* = 132)	GTD group (*n* = 44)	*P* value
Type of ART n (%)			< 0.001
IVF	108 (81.8%)	15 (34.1%)	
ICSI	24 (18.2%)	23 (52.3%)	
PGT	0 (0.0%)	6 (13.6%)	
Total gonadotrophin-releasing hormone agonist dose (IU) ^a^	0.9 (0.0–3.8)	1.2 (0.0–3.8)	0.407
Days of gonadotropin ^b^	10.0 (7.0–20.0)	10.0 (5.0–18.0)	0.602
Total gonadotropin dose (IU) ^a^	2180.5 ± 714.5	1977.7 ± 897.5	0.135
Triger day			
FSH ^a^	14.2 ± 5.2	12.5 ± 4.2	0.054
E_2_^a^	2907.5 ± 1667.5	3418.8 ± 2262.2	0.111
LH ^a^	2.0 ± 1.6	2.5 ± 2.6	0.123
P ^a^	0.7 ± 0.5	0.7 ± 0.4	0.851
Follicle (14–16 mm) ^b^	9.0 (1.0–20.0)	11.5 (1.0–24.0)	0.239
Triger day endometrial thickness (mm) ^a^	11.5 ± 2.4	9.3 ± 2.5	< 0.001
No. of mature oocytes ^b^	9.0 (1.0–22.0)	10.0 (1.0–23.0)	0.929
Normal fertilization rate ^a^	79%	80%	0.986
No. of degenerating cleavages ^a^	0.1 ± 0.6	0.4 ± 0.7	0.005
No. of good-quality embryos ^a^	4.6 ± 2.6	3.5 ± 3.2	0.019
No. of abandoned embryos ^a^	2.8 ± 2.2	4.0 ± 4.2	0.018

^a^ Values are mean ± standard deviation.

^b^ Values are median and Min-Max.

*P* value: For continuous variables, it can be obtained by Kruskal Wallis rank sum test. If the count variable has a theoretical number < 10, it can be obtained by Fisher’s exact probability test.

### Pregnancy outcomes

In evaluating the implantation rate and ovarian hyperstimulation syndrome (OHSS) rate, there were no statistically significant differences (*p* > 0.05), although slight discrepancies existed between the two groups (implantation rate: 55% vs 48%, OHSS rate: 8.3% vs 13.6%). Fifteen patients (34.1%) in the GTD group and 88 patients (66.7%) in the control group had already delivered, 19 (43.2%) and 27 (20.5%) patients were not pregnant, 3 (6.8%) and 4 (3.0%) patients had miscarriage, and 4 (9.1%) and 8 (6.1%) patients were still in pregnancy, respectively. There was a statistically significant difference in the gestational age at delivery (*p* < 0.01): the mean gestational age of patients in the control group was 38.1 ± 1.6 weeks, and that of patients in the GTD group was 36.3 ± 3.3 weeks. Regarding the mode of delivery, there was no difference in the cesarean delivery rate between the two groups (*p* > 0.05). All the outcomes are presented in Table 4.

**Table 4:** Cycle and delivery outcomes

Characteristics	control group (*n* = 132)	GTD group (*n* = 44)	*P* value
No. of embryos transferred ^a^	1.6 ± 0.5	1.4 ± 0.6	0.059
Implantation rate ^a^	55.2%	47.6%	0.290
OHSS n (%)			0.302
NO	121 (91.7%)	38 (86.4%)	
YES	11 (8.3%)	6 (13.6%)	
Clinical outcomes n (%)			
Not pregnant	27 (20.5%)	19 (43.2%)	0.008
Biochemical pregnancy	2 (1.5%)	3 (6.8%)	0.160
miscarriage	4 (3.0%)	3 (6.8%)	0.445
Ectopic pregnancy	2 (1.5%)	0 (0.0%)	1.000
Termination of pregnancy	1 (0.8%)	0 (0.0%)	1.000
Live birth	88 (66.7%)	15 (34.1%)	0.001
ongoing pregnancy	8 (6.1%)	4 (9.1%)	0.644
Gestational age at delivery (WK) ^a^	38.1 ± 1.6	36.3 ± 3.3	< 0.001
Mode of delivery n (%)			0.267
spontaneous delivery	16 (18.2%)	1 (6.7%)	
cesarean delivery	72 (81.8%)	14 (93.3%)	

^a^ Values are mean ± standard deviation.

^b^ Values are median and Min-Max.

*P* value: For continuous variables, it can be obtained by Kruskal Wallis rank sum test. If the count variable has a theoretical number < 10, it can be obtained by Fisher’s exact probability test.

To identify covariates independently associated with the primary outcome of live birth, we conducted multivariate logistic regression (Table 5). After adjusting for age, infertility duration, gravidity, number of curettages, endometrial thickness on trigger day, number of good-quality embryos and number of abandoned embryos, we found that only the number of good-quality embryos (≥3) was associated with the live-birth rate (OR: 2.1; 95% CI: 1.0–4.2, *p* < 0.01). We also found that some factors were also related to the live-birth rate (not statistically significant), such as the age of the patients and endometrial thickness on the trigger day. In the multivariate analysis, the presence of a GTD history did not significantly impact the live-birth rate associated with IVF/ICSI cycles (OR: 0.5; 95% CI: 0.2–1.7, *p* = 0.268). In the GTD group, there were no significant differences in the live-birth rate associated with IVF/ICSI/PGT cycles (Table 6).

**Table 5:** Multivariate logistic regression for live-birth outcome

Covariates	OR (95% CI)	*P* value
Age (year)		
< 35	1.0	Reference
≥ 35	0.5 (0.2, 1.0)	0.065
Infertility duration (year)		
< 3	1.0	Reference
≥ 3	1.1 (0.5, 2.1)	0.828
Gravidity		
< 3	1.0	Reference
≥ 3	0.6 (0.3, 1.4)	0.233
No. of curettage		
< 1	1.0	Reference
≥ 1	0.8 (0.3, 1.7)	0.504
Type of ART		
IVF	1.0	
ICSI	0.9 (0.4, 1.9)	0.733
PGT	1.0 (0.1, 7.5)	0.970
Triger day endometrial thickness (mm)		
≤ 9	1.0	Reference
9–12	2.1 (0.9, 5.1)	0.098
≥ 12	1.9 (0.8, 4.8)	0.165
No. of good-quality embryos		
< 3	1.0	
≥ 3	2.1 (1.0, 4.2)	0.041
No. of abandoned embryos		
< 6	1.0	Reference
≥ 6	1.2 (0.6, 2.6)	0.580
GTD history		
no	1.0	Reference
yes	0.5 (0.2, 1.7)	0.268

**Table 6:** The live birth rate in IVF/ICSI/PGT cycles of patients in GTD group

Type of ART	IVF (*n* = 15)	ICSI (*n* = 23)	PGT (*n* = 6)	*P* value
Live birth rate	33.3%	34.8%	33.3%	0.995

### Immunohistochemical results of endometrial receptivity

**Fig. 2:**
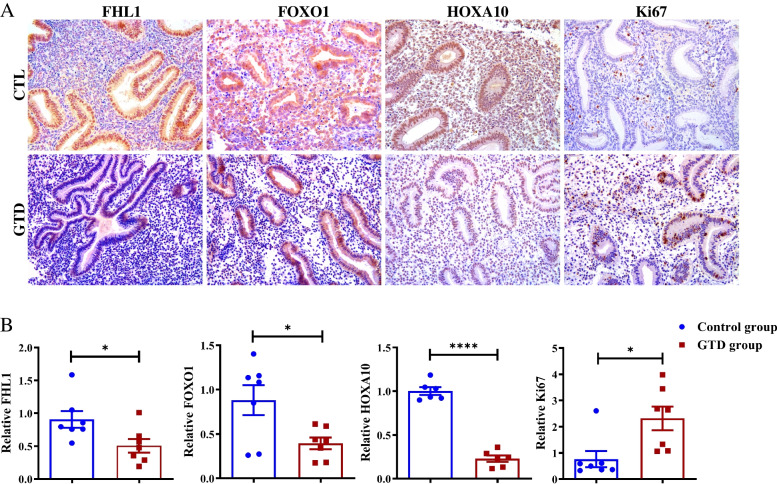
Immunohistochemical results of endometrial receptivity. **A** Representative immunohistochemical endometrium of patients with GTD history or without for FHL1, FOXO1, HOXA10, Ki67. **B** Signal statistics of immunohistochemical staining for FHL1(CTL group n = 7, GTD group n = 7, *p* = 0.0299), FOXO1(CTL group n = 7, GTD group n = 7, *p* = 0.0204), HOXA10(CTL group n = 6, GTD group n = 6, *p* < 0.0001), Ki67 (CTL group n = 7, GTD group n = 7, *p* = 0.0143)

To determine the influence of endometrial factors on the live-birth outcome, endometrial receptivity was measured in some of these patients. As shown by IHC (Fig. 2), HOXA10 and FHL1 expression was significantly lower (*p* < 0.05) in the endometrial epithelial and stromal cells of infertile women with a GTD history than in those of the controls. FOXO1 expression was evidently lower (*p* < 0.05), mainly in stromal cells. Ki67 expression was much higher (*p* < 0.05) in both endometrial epithelial and stromal cells of patients with a GTD history.

## Discussion

Gestational trophoblastic disease, especially hydatidiform mole, is thought to be associated with empty oocyte fertilization or polyspermy [17, 18]. In this retrospective cohort study of 176 patients undergoing IVF/ICSI cycles for infertility, a history of GTD was associated with embryologic outcomes, including a higher ratio of ICSI/PGT cycles, fewer good-quality embryos and more abandoned embryos, but it did not statistically impact the fertilization rate or normal fertilization rate. In addition, the GTD group had a higher rate of miscarriage, which might be related to poor embryo quality. Studies have shown that NLRP7, C6ORF221 and other gene mutations can lead to recurrent hydatidiform mole and oocyte defects [19, 20]. In the conventional IVF/ICSI process, embryo quality is evaluated mainly by morphology, which cannot objectively evaluate the embryo’s subsequent developmental potential. In this study, we found that patients with a GTD history had fewer good-quality embryos and more abandoned embryos, which might be attributed to some of them undergoing PGT. It has been reported that a high proportion of aneuploidy of embryos from patients with homozygous mutations in NLRP7 was detected through PGT [20]. Our statistical results showed that neither ICSI nor PGT improved the live-birth rate of patients with a history of GTD (Table 6). Therefore, it is worth considering whether the PGT technique is needed for patients with recurrent GTD.

In addition to embryological factors, the endometrium must also be considered. A thin endometrium on ultrasound in the course of ovarian hyperstimulation has been thought to be associated with pregnancy rates in IVF/ICSI [21]. In our study, the endometrial thickness of patients with a GTD history was much thinner than that of the control patients. A thin endometrium can result in impaired endometrial receptivity, leading to reduced embryo implantation and pregnancy rates in IVF/ICSI [22, 23].

Blastocysts were successfully implanted only when transferred into the receptive uterus [24]. In humans, the pre-receptive phase spans 7 days after ovulation (early luteal phase), and endometrial receptivity is achieved in the mid-luteal phase (~ 7–10 days after ovulation). The uterus then proceeds to the nonreceptive phase for the remainder of the cycle (late luteal phase) until the following menstruation ensues [24, 25]. Physiological and molecular processes of endometrial receptivity are complex but highly organized. HOXA10, FOXO1 and FHL1 play an important role in endometrial receptivity and subsequent implantation events [26–28]. As a marker of the proliferation of endometrial glandular and stromal cells, normal Ki67 expression patterns are critical for endometrial receptivity and decidualization [28–30]. Our immunohistochemical results showed that the expression levels of HOXA10, FOXO1 and FHL1 were significantly lower in the endometrium of women with a GTD history and that the expression level of Ki67 was abnormal. These results suggested that the thinner endometrium caused by uterine curettage of women with a GTD history impacted endometrial receptivity, which might be a critical cause of lower implantation and pregnancy rates.

At present, many studies of subsequent natural pregnancy in GTD patients have found that, except for the increased risk of stillbirth and premature birth, most pregnancy conditions are not significantly different from those in patients without a GTD history [5, 31]. However, it should not be ignored that approximately 4.4% of these GTD patients still have secondary infertility, and there are few reports on these secondary infertile patients who turn to IVF/ICSI cycles for successful pregnancy. According to the data of our center, the number of infertile patients with a GTD history in the last 5 years was 44, and the main reason for infertility was fallopian tube factor (54.5%). Although the live-birth rate and gestational age at delivery in these patients were lower than those in patients without a history of GTD, there were no significant differences in the incidence rate of OHSS and cesarean delivery between the two groups, which was quite similar to the natural pregnancy in GTD patients. This finding suggests that patients who are able to have a successful pregnancy after IVF/ICSI treatment need to be more aware of an increased risk of miscarriage and preterm birth.

Similarly, we also need to consider whether ovulation induction drugs will increase the probability of subsequent progression to recurrent GTD in these patients. Patignat et al. [32] retrospectively analyzed the data of 52 patients who developed hydatidiform mole after treatment with clomiphene or Gn ovulation induction, and the results showed that the probability of subsequent progression of GTD in these patients was not significantly different from that of patients who got hydatidiform mole after a natural pregnancy. In addition, the total dosage of drugs was similar in the two groups. Therefore, ovulation stimulation drugs should not be a critical factor for patients with recurrent GTD.

The disadvantage of this retrospective study is that the sample size was small, which may be related to the decreasing incidence of GTD in recent years [33] and the low secondary infertility rate after GTD [34–36]. There are still some patients who have not received embryo transfer, and further follow-up of these patients is needed.

In conclusion, secondary infertile patients with a history of GTD can obtain similar numbers of normal fertilization in the process of IVF/ICSI cycles as patients without a history of GTD. However, the pregnancy rate is lower than that of patients without a GTD history due to two factors: (1) the fewer available embryos and more abandoned embryos and (2) the thinner endometrial thickness because of the greater number of uterine curettages. Furthermore, the miscarriage rate was higher and the gestational age at delivery was smaller, which might result from the harmful effect of uterine curettage. This study can provide some clinical data for IVF/ICSI cycles in infertile patients with a history of GTD to better guide clinical treatment.

## Supplementary Information

**Additional file 1: Supplemental Table 1.** The reasons of patients receiving hysteroscopic in Control group and GTD group.

## Data Availability

The datasets used or analyzed during the current study are available from the corresponding author on reasonable request.
