# Supplementary material for: Association between gestational trophoblastic disease (GTD) history and clinical outcomes in in vitro fertilization/intracytoplasmic sperm injection (IVF/ICSI) cycles
Source: Reprod Biol Endocrinol. 2022 Feb 4;20:27. doi: 10.1186/s12958-022-00898-2 (PMC8815202; doi:10.1186/s12958-022-00898-2)
Supplement: Supplementary file 1 — Additional file 1: Supplemental Table 1. The reasons of patients receiving hysteroscopic in Control group and GTD group. [file 12958_2022_898_MOESM1_ESM.docx]

Supplemental Table1. The reasons of patients receiving hysteroscopic in Control group and GTD group

| Reason | Control group  (n=7) | GTD group  (n=7) |
| --- | --- | --- |
| Curettage history | 1 | 3 |
| Suspected polyp | 3 | 2 |
| Suspected adhesion | 2 | 2 |
| Other reasons attending  physicians consider necessary | 1 | 0 |
